# Urinary and circulatory levels of plasticizer metabolites and their associations with renal function: a cross-sectional analysis of the NHANES cohort

**DOI:** 10.1080/07853890.2025.2559125

**Published:** 2025-10-05

**Authors:** Yuxiang Liu, Linhao Zhang, Chujie Sun, Huifang Wang, Yaxiong Guo, Ruiqin Xue, Yating Xing

**Affiliations:** a Department of Nephrology, Shanxi Provincial People’s Hospital (The Fifth Clinical Medical College of Shanxi Medical University), Taiyuan, China; b First Clinical College, Shanxi University of Chinese Medicine, Jinzhong, Shanxi, China; c Shanxi Provincial Key Laboratory of Kidney Disease, Taiyuan, China; d Faculty of Health and Wellness, City University of Macau, Macau, China; e Department of Basic Medicine, Fenyang College of Shanxi Medical University, Fenyang, China; f The First Clinical Medical School, Shanxi Medical University, Taiyuan, China; g Department of Surgical Unit 1, Shanxi Combined Traditional Chinese and Western Medicine Hospital, Taiyuan, China; h Fifth Clinical College, Shanxi Medical University, Taiyuan, China; i Texas State University, San Marcos, Texas, USA

**Keywords:** Bayesian kernel machine regression, chronic kidney disease(CKD), phthalates, plasticizers, renal function

## Abstract

**Background:**

Plasticizers used in consumer products raise concerns for nephrotoxicity; while DEHP is well characterized, evidence for DEHTP and DiNP—particularly their metabolites—remains limited. We examined associations of five urinary metabolites—MEHHP/MECPP (DEHP), MEHHTP/MECPTP (DEHTP), and MONP (DiNP)—with renal endpoints in a nationally representative adult sample.

**Methods:**

We analyzed 2,229 adults from NHANES 2015–2018 using multivariable linear and restricted cubic spline models for eGFR, and evaluated combined-exposure effects with WQS and BKMR; analyses were repeated with UACR as a secondary outcome.

**Results:**

Higher MECPP and MEHHP were associated with lower eGFR, whereas positive associations for MECPTP and MEHHTP may reflect early glomerular hyperfiltration rather than benefit. As a secondary endpoint, UACR indicated early injury: MECPP increased UACR (*p* < 0.001) and MONP decreased UACR (*p* = 0.002), with mild nonlinearity. Combined-exposure models prioritized DEHP/DEHTP metabolites, with MECPTP contributing most.

**Conclusion:**

Urinary plasticizer metabolites are linked to altered renal endpoints. Heterogeneous exposure–response patterns support complementary outcomes and mixture methods; longitudinal and mechanistic studies are needed to clarify causality and inform risk management for emerging plasticizers.

## GRAPHICAL ABSTRACT

**Figure:**
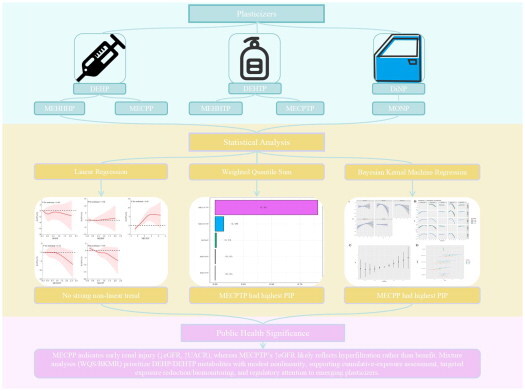

## 1. Background

Plasticizers are chemical compounds added to polymers to enhance flexibility and durability. These compounds are used in a wide array of products, from building materials (e.g. vinyl flooring,adhesives) and consumer goods (detergents, lubricants, plastics, synthetic fabrics) to personal care items (soaps, shampoos, hairsprays, nail polish) and medical supplies (blood storage bags and tubing). As a result, people are often unknowingly exposed to plasticizers in daily life [1].

Once absorbed, plasticizers are quickly hydrolyzed to monoesters, which may undergo further oxidation depending on the parent compound [2–4]. These metabolites can be conjugated *via* glucuronidation and are primarily excreted in urine and feces [5]. Di(2-ethylhexyl) phthalate (DEHP) is the most widely used plasticizer. Two significant urinary biomarkers of DEHP exposure—mono(2-ethyl-5-hydroxyhexyl) phthalate (MEHHP) and mono(2-ethyl-5-carboxypentyl) phthalate (MECPP)—collectively account for approximately 42% of excreted DEHP (MEHHP: 23.3%, MECPP: 18.5%) [6]. Animal studies indicate that these metabolites induce reproductive and developmental toxicity [7–11].

DEHP use is declining, mainly due to U.S. and EU regulations limiting its use in toys and food-contact materials [12]. National biomonitoring data showed a 37% decline in adjusted geometric mean concentrations of DEHP metabolites from 2001 to 2002 [13]. In contrast, the utilization of alternative plasticizers such as diisononyl phthalate (DiNP) and di(2-ethylhexyl) terephthalate (DEHTP)—structural analogs and functional substitutes of DEHP—has likely been increasing [13,14]. DEHTP is a versatile plasticizer widely used in polymers, coatings, adhesives, and sealants, and it is found in products such as flooring, cable insulation, toys, medical devices, and food packaging [12]. As exposure to DEHTP increases, there is growing concern about its potential health effects. MEHHTP and MECPTP, two oxidative metabolites of DEHTP, are established as reliable biomarkers of DEHTP exposure [15–18]. Combined, these two metabolites account for roughly 15% of an ingested DEHTP dose excreted in urine, with MECPTP alone contributing about 13% [15]. All other DEHTP metabolites represent under 1% of excreted DEHTP [17]. Since the early 2000s, DEHP biomarkers (e.g. MEHHP, MECPP) have been detected more frequently in the general population, suggesting a shift in exposure toward DEHTP [15–19]. DiNP, another high-molecular-weight phthalate, is used in numerous industrial, commercial, and consumer products (e.g. automotive care items, fuels, construction materials, electrical cables, adhesives, paints, electronics, children’s toys) [20]. The U.S. Environmental Protection Agency (EPA) recently endorsed the continued use of DiNP in most applications, likely facilitating its further integration into everyday products. Preliminary toxicological data suggest that DiNP’s primary metabolite, mono-oxoisononyl phthalate (MONP), has lower reproductive and endocrine-disrupting potential than DEHP metabolites [21]. However, limited biomonitoring data on these emerging plasticizers means the human health implications of DEHP alternatives like DiNP and DEHTP remain poorly understood, warranting urgent investigation.

Oxidative stress and inflammation are central processes in many chronic diseases, including cardiovascular disease, cancer, chronic kidney disease (CKD), and neurodegenerative conditions. Understanding how plasticizer metabolite exposure affects these pathways is crucial for developing targeted prevention and treatment strategies, especially as environmental pollution escalates.

CKD is a pressing global public health concern. CKD affects an estimated 697.5 million people worldwide, corresponding to a global prevalence of 9.1%. The disease often progresses to end-stage renal disease (ESRD), requiring dialysis or transplantation, and it ranks among the top 12 causes of death worldwide [22]. CKD imposes significant economic and psychological burdens on individuals and healthcare systems.

CKD has a multifactorial etiology, involving well-established factors (e.g. metabolic disorders, immune dysfunction, genetic susceptibility) and increasingly recognized environmental determinants [23]. With CKD prevalence rising and many risk factors, clarifying the role of environmental pollutants, including plasticizers, is paramount.

While the adverse renal effects of traditional phthalates (PAEs) are well documented, the health impacts of newer plasticizers like DiNP and DEHTP remain under-studied—particularly their roles in oxidative stress and inflammation, key mechanisms in CKD pathogenesis. Furthermore, other environmental toxicants—including delicate particulate matter (PM_2_._5_), heavy metals, and microplastics—have increasingly been implicated in kidney function deterioration [24–26], raising concern about the cumulative impact of long-term exposures.

In response to these concerns, we used U.S. nationally representative data to examine the relationships between urinary biomarkers of DEHTP, DEHP, and DiNP and kidney function markers. Specifically, we examined five metabolites—MEHHTP and MECPTP (DEHTP metabolites), MONP (a DiNP metabolite), and MEHHP and MECPP (DEHP metabolites). Using statistical models—including Bayesian Kernel Machine Regression (BKMR)—we assessed nonlinear and interactive effects of multiple plasticizer metabolites on estimated glomerular filtration rate (eGFR), a key indicator of renal function.

This study provides an environmental health perspective and robust evidence to inform targeted public health interventions and regulatory strategies. Our goal is to help reduce the nephrotoxic burden of plasticizer exposure, especially given rising environmental contamination.

## 2. Materials and methods

### 2.1. Study population

Because urinary biomarkers of DEHTP were only measured beginning in the 2015 National Health and Nutrition Examination Survey (NHANES) cycle, we utilized data from NHANES 2015–2018. We included participants aged 18 and older with complete data on urinary plasticizer metabolites, blood-based oxidative stress and inflammation markers, and relevant covariates. After applying exclusion criteria (see Supplementary Figure 1), 2,229 adults were included in the final analysis.

This study was conducted in accordance with the ethical principles outlined in the Declaration of Helsinki. The NHANES study protocols were approved by the National Center for Health Statistics (NCHS) Research Ethics Review Board (Protocol #2011-17 & Protocol #2018-01), and all participants provided written informed consent prior to data collection.

### 2.2. Assessment of renal function

Serum creatinine levels were measured using the Jaffe rate method. Calibration was performed using the following equation:

$${\text{eGFR}}_{\text{MDRD}}\text{=175}{\text{.0*Scr}}^{-\text{1.154}}*{\text{age}}^{-\text{0.203}}\text{*}0.742\left(\text{if female}\right)\text{*}1.\text{212(if black)}$$

where *x* is the uncalibrated serum creatinine concentration.

The estimated glomerular filtration rate (eGFR) was calculated using both the modified 4-variable Modification of Diet in Renal Disease (MDRD) Study equation [27] and the Chronic Kidney Disease Epidemiology Collaboration (CKD–EPI) equation [28]:

$${\text{eGFR}}_{\text{ckd}‐\text{EPI}}\text{=141*min }{\left(\frac{\text{Scr}}{k}\text{,1}\right)}^{a}\text{*max}{\left(\frac{\text{Scr}}{k}\text{,1}\right)}^{-\text{1.029}}*0{\text{.993}}^{\text{age}}\text{*}1.108\left(\text{if}\;\text{female}\right)\text{*}1.\text{159(}\text{if}\;\text{black}\text{)}$$

In the CKD–EPI equation, κ is 0.7 for females and 0.9 for males, and α is −0.329 for females and −0.411 for males.

### 2.3. Measurement of plasticizer metabolites

Urine samples were transported in cryogenic containers and packaged securely with dry ice to minimize degradation. To preserve sample integrity, prolonged exposure to room or refrigerated temperatures was avoided. All specimens were stored at −40 °C or lower until analysis.

The concentrations of plasticizer metabolites in urine were quantified using high-performance liquid chromatography coupled with electrospray ionization tandem mass spectrometry (HPLC–ESI–MS/MS). During sample processing, glucuronidated metabolites were hydrolyzed enzymatically, followed by online solid-phase extraction (SPE) and reverse-phase HPLC–ESI–MS/MS analysis. Isotopically labelled internal phthalate metabolites and MHNCH standards were included to enhance analytical precision. For values below the limit of detection (LOD), imputed estimates using the lowest detectable concentration were applied.

### 2.4. Covariates

We adjusted for a range of covariates with clinical and epidemiological relevance, including age, sex, body mass index (BMI), educational attainment, marital status, family poverty income ratio (PIR), alcohol consumption, smoking status, diabetes, and hypertension.

BMI was calculated as weight (kg) divided by height squared (m^2^). PIR was categorized into low (PIR ≤1.30), medium (1.30 < PIR ≤3.50), and high-income (PIR > 3.50) groups. Alcohol consumption was classified as never (0 g/day), moderate (0.1–27.9 g/day for men; 0.1–13.9 g/day for women), and heavy (>28 g/day for men; >14 g/day for women). Smoking status was defined based on lifetime consumption of ≥100 cigarettes.

Hypertension was defined as self-reported use of antihypertensive medications, systolic blood pressure ≥140 mmHg, or diastolic blood pressure ≥90 mmHg. Diabetes was defined as hemoglobin A1c (HbA1c) ≥6.5% or self-reported current use of insulin or oral hypoglycemic agents.

### 2.5. Statistical analysis

Descriptive statistics were used to characterize the study population. Continuous variables were presented as means with standard deviations (SD), while categorical variables were expressed as counts and percentages. Urinary plasticizer metabolite concentrations were log_10_-transformed to normalize their distributions [29]. Pearson correlation analysis assessed the associations between metabolite levels and eGFR.

First, linear regression models were applied to evaluate the individual associations between each plasticizer metabolite and eGFR. Restricted cubic spline models were then used to assess potential nonlinear exposure–response relationships.

Second, we employed Weighted Quantile Sum (WQS) regression to assess the effects of multiple plasticizer exposures on renal function. WQS constructs a weighted index of correlated exposures and estimates their combined effect on the outcome. This study used 10,000 bootstrap iterations to construct WQS indices in both positive and negative directions. When the WQS index showed statistical significance, corresponding weights were examined to determine the relative contribution of each metabolite. The dataset was randomly partitioned, with 40% of observations assigned to the training set and 60% to the validation set [30,31].

Third, we applied Bayesian Kernel Machine Regression (BKMR) to explore plasticizer mixtures’ joint and individual effects on eGFR within a Bayesian variable selection framework. BKMR allows flexible modelling of complex, nonlinear, and interactive relationships among co-exposures. Posterior inclusion probabilities (PIPs) were calculated to assess the relative importance of each metabolite, with a threshold of 0.5 used to denote strong evidence of association. Both univariate and bivariate exposure–response functions were generated while fixing remaining exposures at selected percentiles (25th, 50th, and 75th). BKMR models were run using Markov Chain Monte Carlo (MCMC) algorithms with 10,000 iterations [32].

Fourth, we conducted interaction tests using the Cross-Validated Ensemble of Kernels (CVEK) approach to evaluate pairwise interactions between plasticizer metabolites about eGFR [33].

Finally, sensitivity analyses were performed using unadjusted (crude) linear models and single-pollutant models (adjusted for all covariates but including one metabolite at a time) to examine the robustness of the findings.

## 3. Results

### 3.1. Descriptive statistics

Nineteen thousand two hundred twenty-five participants were initially recruited from the NHANES 2015–2018 cycle. As illustrated in Supplementary Figure 1, individuals were sequentially excluded due to missing data on serum creatinine (*n* = 7,067), urinary MEHHP (*n* = 8,270), MEHHTP (*n* = 302), education level (*n* = 596), poverty income ratio (PIR, *n* = 338), height and weight (*n* = 87), alcohol consumption (*n* = 120), hypertension status (*n* = 213), and diabetes status (*n* = 3). Additionally, no participants aged under 18 years were retained (*n* = 0). After applying all exclusion criteria, 2,229 adult participants with complete covariate and exposure data were included in the final analysis.

As shown in Table 1, the study population (*N* = 2,229) had a mean age of 49.77 years, with a balanced sex ratio and diverse racial/ethnic composition. Most participants had attained at least a high school education, and the prevalence of hypertension (40.9%) and diabetes (17.2%) was consistent with general population estimates.

**Table 1.** Baseline demographic and clinical characteristics of participants in NHANES 2015–2018 (*N* = 2,229).

Variable	Total (*N* = 2229)	2015–2016 (*n* = 1111)	2017–2018 (*n* = 1118)
Age, mean ± SD (years)	49.77 ± 17.47	49.07 ± 17.31	50.47 ± 17.61
Sex, *n* (%)			
Male	1109 (49.75)	538 (48.42)	571 (51.07)
Female	1120 (50.25)	573 (51.58)	547 (48.93)
Education level, *n* (%)			
<High school	401 (17.99)	207 (18.63)	194 (17.35)
High school graduate	508 (22.79)	260 (23.40)	248 (22.18)
Some college or above	1320 (59.22)	644 (57.97)	676 (60.47)
Race/Ethnicity, *n* (%)			
Mexican American	309 (13.86)	175 (15.75)	134 (11.99)
Other Hispanic	241 (10.81)	147 (13.23)	94 (8.41)
Non-Hispanic White	841 (37.73)	401 (36.09)	440 (39.36)
Non-Hispanic Black	494 (22.16)	248 (22.32)	246 (22.00)
Asian	240 (10.77)	93 (8.37)	147 (13.15)
Other/Mixed	104 (4.67)	47 (4.23)	57 (5.10)
Marital status, *n* (%)			
Married	1148 (51.50)	581 (52.30)	567 (50.72)
Never married	413 (18.53)	210 (18.90)	203 (18.16)
Separated/Divorced/Widowed	668 (29.97)	320 (28.80)	348 (31.13)
PIR (Poverty Income Ratio), *n* (%)			
Low (≤1.30)	630 (28.26)	319 (28.71)	311 (27.82)
Medium (1.30–3.50)	902 (40.47)	444 (39.96)	458 (40.97)
High (>3.50)	697 (31.27)	348 (31.32)	349 (31.22)
Body mass index (kg/m²)	28.89 ± 6.75	28.85 ± 6.49	28.93 ± 7.00
Smoking status, *n* (%)			
Yes	977 (43.83)	494 (44.46)	483 (43.20)
No	1252 (56.17)	617 (55.54)	635 (56.80)
Alcohol consumption, *n* (%)			
Never	1718 (77.07)	851 (76.60)	867 (77.55)
Moderate	151 (6.77)	75 (6.75)	76 (6.80)
Heavy	360 (16.15)	185 (16.65)	175 (15.65)
Hypertension, *n* (%)			
Yes	912 (40.92)	453 (40.77)	459 (41.06)
No	1317 (59.08)	658 (59.23)	659 (58.94)
Diabetes, *n* (%)			
Yes	384 (17.23)	176 (15.84)	208 (18.60)
No	1845 (82.77)	935 (84.16)	910 (81.40)
Urinary metabolites, mean ± SD (log₁。 transformed)			
MEHHP	0.71 ± 0.45	0.76 ± 0.45	0.66 ± 0.44
MECPP	0.90 ± 0.44	0.94 ± 0.44	0.86 ± 0.43
MEHHTP	0.68 ± 0.67	0.62 ± 0.67	0.75 ± 0.67
MECPTP	1.23 ± 0.72	1.16 ± 0.72	1.30 ± 0.72
MONP	0.23 ± 0.50	0.32 ± 0.53	0.14 ± 0.45
eGFR (mL/min/1.73 m²)	100.65 ± 24.14	101.89 ± 23.97	99.42 ± 24.26

Note: Values are presented as mean ± SD for continuous variables and *n* (%) for categorical variables. Urinary metabolite concentrations are log₁。-transformed.

Urinary metabolite levels showed inter-individual variability, with MECPTP presenting the highest mean concentration, while MONP was the lowest. Slight differences across survey cycles were noted, particularly in MEHHTP and MONP, suggesting possible temporal exposure variation. The average eGFR was above 100 mL/min/1.73 m^2^, indicating preserved renal function in the cohort. These baseline characteristics provide essential context for interpreting subsequent association analyses.

### 3.2. Correlation analysis

**Figure 1.**
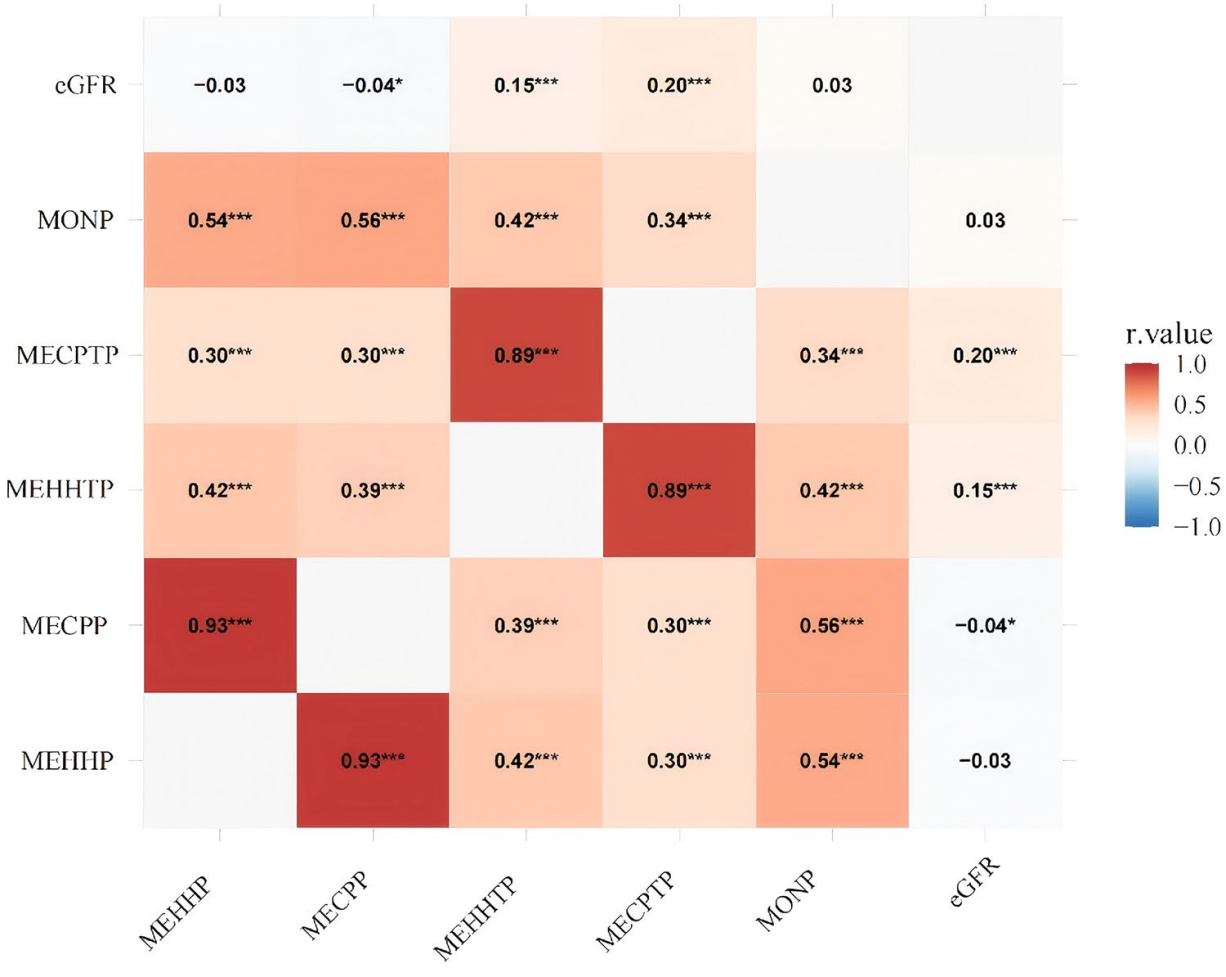
Pearson correlation matrix between urinary plasticizer metabolites and estimated glomerular filtration rate (eGFR). Pearson correlation matrix among urinary plasticizer metabolites and eGFR (two-tailed tests; ***p < 0.001, **p < 0.01, *p* < 0.05 Spearman’s correlations including UACR are provided in Figure S2.

In the Pearson correlation analysis, the strength of the association between exposure variables and renal function outcomes varied across the dataset. Notably, a strong positive correlation was observed among several plasticizer metabolites, suggesting shared metabolic or environmental exposure pathways. Most pairwise correlations were statistically significant and optimistic among the six variables examined—MEHHP, MECPP, MEHHTP, MECPTP, MONP, and eGFR.As illustrated in Figure 1, these correlations highlight distinct clustering patterns among DEHP- and DEHTP-derived metabolites.

The correlation between MECPP and MEHHP was exceptionally high (*r* = 0.93, *p* < 0.001), indicating substantial collinearity, likely due to their shared parent compound (DEHP). Similarly, MEHHTP and MECPTP—both DEHTP metabolites—exhibited a strong correlation (*r* = 0.89, *p* < 0.001). MONP was moderately correlated with MECPP (*r* = 0.56, *p* < 0.001), and a weaker but significant association was found between MEHHP and MEHHTP (*r* = 0.42, *p* ≈ 3.04 × 10–96). Additional positive correlations were observed between MEHHP and MECPTP (*r* = 0.30, *p* < 0.001), MEHHTP and MONP (*r* = 0.42, *p* < 0.001), and MECPTP and MONP (*r* = 0.34, *p* < 0.001).

In contrast, eGFR was only weakly associated with most metabolites. It was negatively correlated with MEHHP (*r* = –0.03, *p* = 0.125) and positively with MONP (*r* = 0.03, *p* = 0.139), though neither reached statistical significance. However, eGFR exhibited a modest but statistically significant negative correlation with MECPP (*r* = –0.04, *p* = 0.045), and stronger positive correlations with MEHHTP (*r* = 0.15, *p* < 0.001) and MECPTP (*r* = 0.20, *p* < 0.001), suggesting potential biological relevance.

### 3.3. Linear regression analysis

In multivariable linear models, urinary MECPP was inversely associated with renal function: each 1 ng/mL increase corresponded to a 4.45 mL/min/1.73 m^2^ lower eGFR (95% CI: −8.47 to–0.42), consistent with adverse kidney function. By contrast, MECPTP showed a positive association with eGFR (β per 1 ng/mL = 2.01 mL/min/1.73 m^2^; 95% CI: 0.10 to 3.93). Given that elevations in eGFR can reflect glomerular hyperfiltration in early nephropathy, this positive association should not be interpreted as renal benefit but rather as a hemodynamic change that warrants cautious interpretation. Full adjusted estimates are reported in Table 2.

**Table 2.** Linear regression estimates of associations between urinary plasticizer metabolites and eGFR.

Variable	β	95% CI (Low)	95% CI (Up)	*p* value
MONP	0.12	−1.45	1.68	0.88
MEHHP	2.36	−1.48	6.20	0.23
MECPP	−4.45	−8.47	−0.42	0.03
MEHHTP	−1.20	−3.32	0.93	0.27
MECPTP	2.01	0.10	3.93	0.04

Note: Linear regression estimates the change in eGFR (mL/min/1.73 m²) per 1 ng/mL increase in each urinary metabolite. CI: confidence interval.

### 3.4. Nonlinear regression analysis

**Figure 2.**
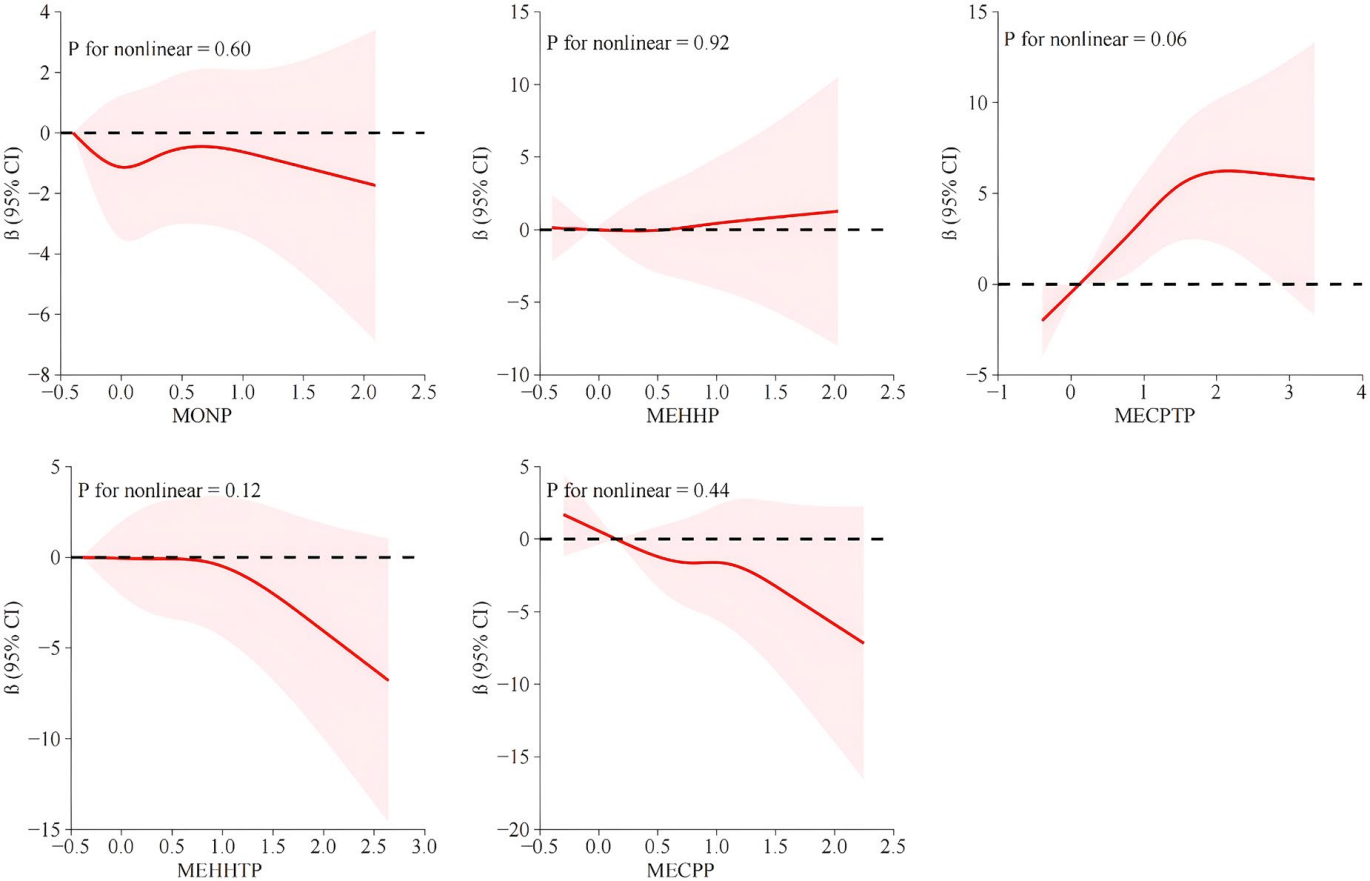
Nonlinear exposure–response relationships between urinary plasticizer metabolites and estimated glomerular filtration rate (eGFR). Restricted cubic spline models illustrate the dose–response associations between each urinary plasticizer metabolite (MEHHP, MECPP, MEHHTP, MECPTP, and MONP) and eGFR. The solid red line represents the point estimate of the association, while the shaded gray area indicates the 95% confidence interval (CI). The *p* value for non-linearity is reported in each panel to assess whether the relationship deviates significantly from linearity. Metabolite concentrations were log₁。-transformed prior to analysis, and all models were adjusted for relevant demographic, socioeconomic, and clinical covariates. Overall, the exposure–response curves predominantly demonstrated linear trends without strong evidence of threshold or saturation effects. For comparison, analogous spline curves depicting associations with urinary albumin-to-creatinine ratio (UACR) are provided in Supplementary Figure S3.

We employed restricted cubic spline models to investigate potential nonlinear associations between plasticizer metabolites and renal function. The exposure–response curves demonstrated predominantly linear patterns across the full spectrum of metabolite concentrations. No substantial evidence of nonlinear threshold effects or saturation points was observed. As illustrated in Figure 2, the relationship between urinary metabolite levels and eGFR appeared to follow consistent trends, reinforcing the findings derived from linear regression models.

### 3.5. Combined exposure analysis

The results of Bayesian Kernel Machine Regression (BKMR) analysis offered insight into the relative importance of individual plasticizer metabolites to the estimated glomerular filtration rate (eGFR). The Posterior Inclusion Probability (PIP) values revealed substantial heterogeneity across compounds. Specifically, MECPP exhibited the highest relevance, with a PIP of 0.99, suggesting a strong and consistent association with decreased eGFR. MECPTP followed with a PIP of 0.85, indicating a robust positive influence. MEHHTP also demonstrated notable importance (PIP = 0.67), while MEHHP (PIP = 0.19) and MONP (PIP = 0.01) were associated with marginal or negligible effects on renal function.

**Figure 3.**
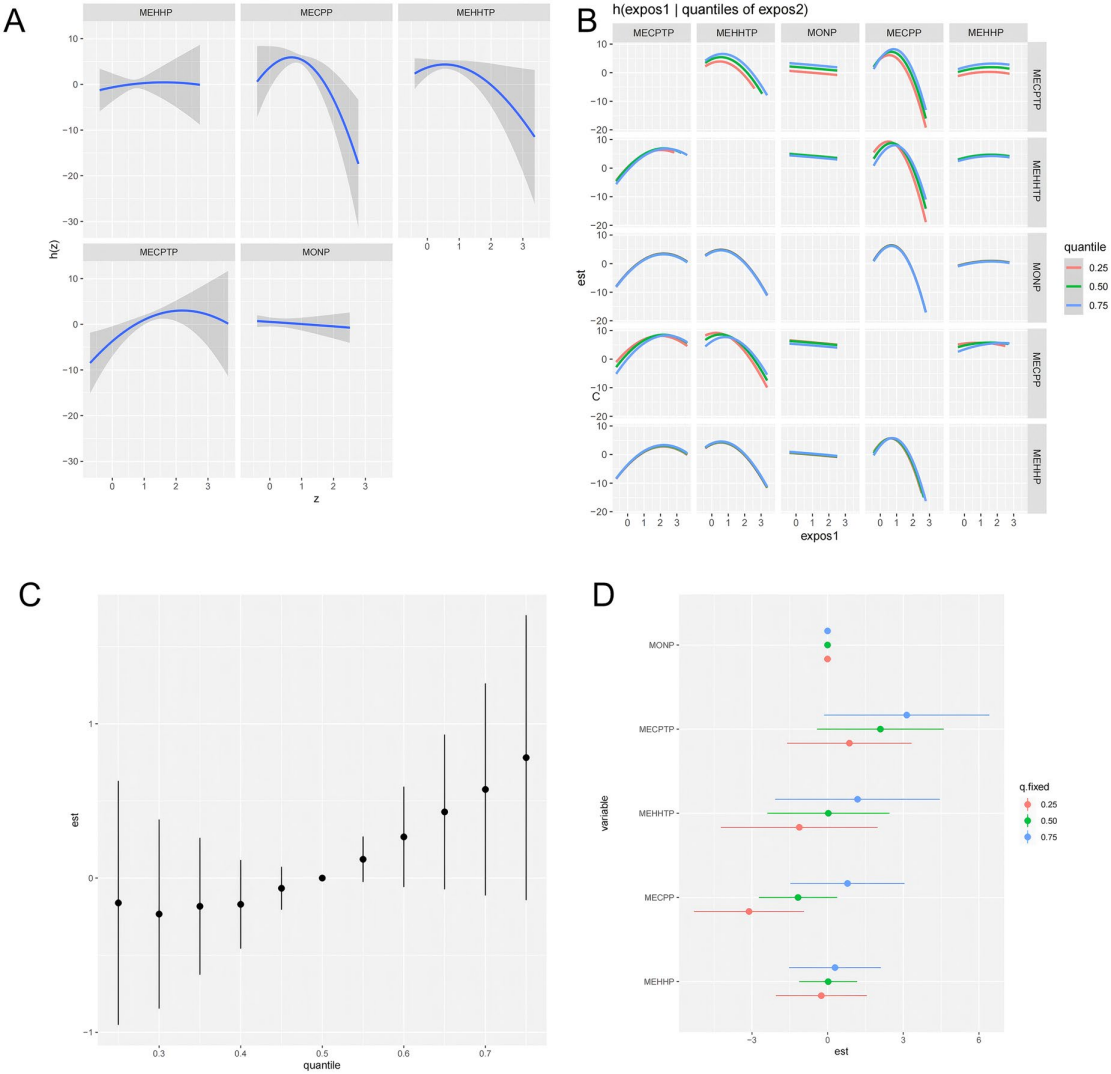
BKMR analysis of urinary plasticizers and eGFR. Bayesian Kernel Machine Regression (BKMR) results depicting the associations of five urinary metabolites—MEHHP and MECPP (DEHP), MEHHTP and MECPTP (DEHTP), and MONP (DiNP)—with estimated glomerular filtration rate (eGFR). (A) Univariate exposure–response functions for each metabolite with the other four exposures fixed at their sample medians; solid line shows the posterior mean and the shaded band the 95% credible interval (CrI). The *x* axis displays metabolite concentrations (log₁。-transformed), and the *y* axis the predicted change in eGFR (mL/min/1.73 m²) relative to the median-exposure reference. (B) Bivariate exposure–response functions illustrating pairwise relationships: for the focal metabolite on the *x* axis, curves are plotted while fixing a second metabolite at the 25th, 50th, or 75th percentile; the remaining three exposures are held at their medians. (C) Overall mixture effect, estimated as the change in eGFR when jointly shifting all five metabolites across exposure percentiles (e.g., 25th → 75th) compared with the median mixture. (D) Single-exposure effects under different mixture backgrounds, obtained by fixing the other four metabolites at the 25th, 50th, or 75th percentile. All BKMR models applied the same covariate adjustments as the main regression analyses and were fit using 10,000 MCMC iterations; Posterior Inclusion Probabilities (PIPs) are reported in the main text. Analogous BKMR results for the urinary albumin-to-creatinine ratio (UACR) are presented in Supplementary Figure S4.

Single-exposure response functions were plotted while holding the remaining four metabolites at their median levels to visualize marginal effects. As shown in Figure 3A, MECPP exhibited a nonlinear dose–response curve, characterized by an initial rise in eGFR followed by a marked decline at higher concentrations, accompanied by increasingly wide confidence intervals. In contrast, MEHHTP displayed a downward trend across its exposure range, while MECPTP was positively associated with eGFR. MEHHP and MONP showed flat trajectories, indicating little to no effect.

To further explore potential interactions, we conducted bivariate visualizations by fixing one metabolite at the 20th, 50th, and 75th percentiles and plotting the dose–response relationship of another metabolite while holding the remaining three at their medians. As illustrated in Figure 3B, the exposure**–**response curves appeared essentially parallel, suggesting minimal interactive effects among the five metabolites.

Overall mixture effects were estimated by jointly shifting all five metabolites to selected percentiles and contrasting the predicted eGFR with the median-exposure scenario. As shown in Figure 3C, the combined-exposure index displayed a modest positive deviation in eGFR that did not reach statistical significance. Because elevations in eGFR may reflect glomerular hyperfiltration in early kidney injury, this pattern should be interpreted cautiously and not as evidence of renal benefit.

Lastly, stratified analyses were performed to assess how the influence of each metabolite varied across different combined backgrounds (i.e.when the other four compounds were fixed at the 25th, 50th, and 75th percentiles). In most cases, effect magnitudes increased with higher background exposure levels. Notably, MECPP displayed a pronounced negative effect when all other metabolites were fixed at the 25th percentile. MONP, however, remained relatively inert across all background conditions (Figure 3D).

**Figure 4.**
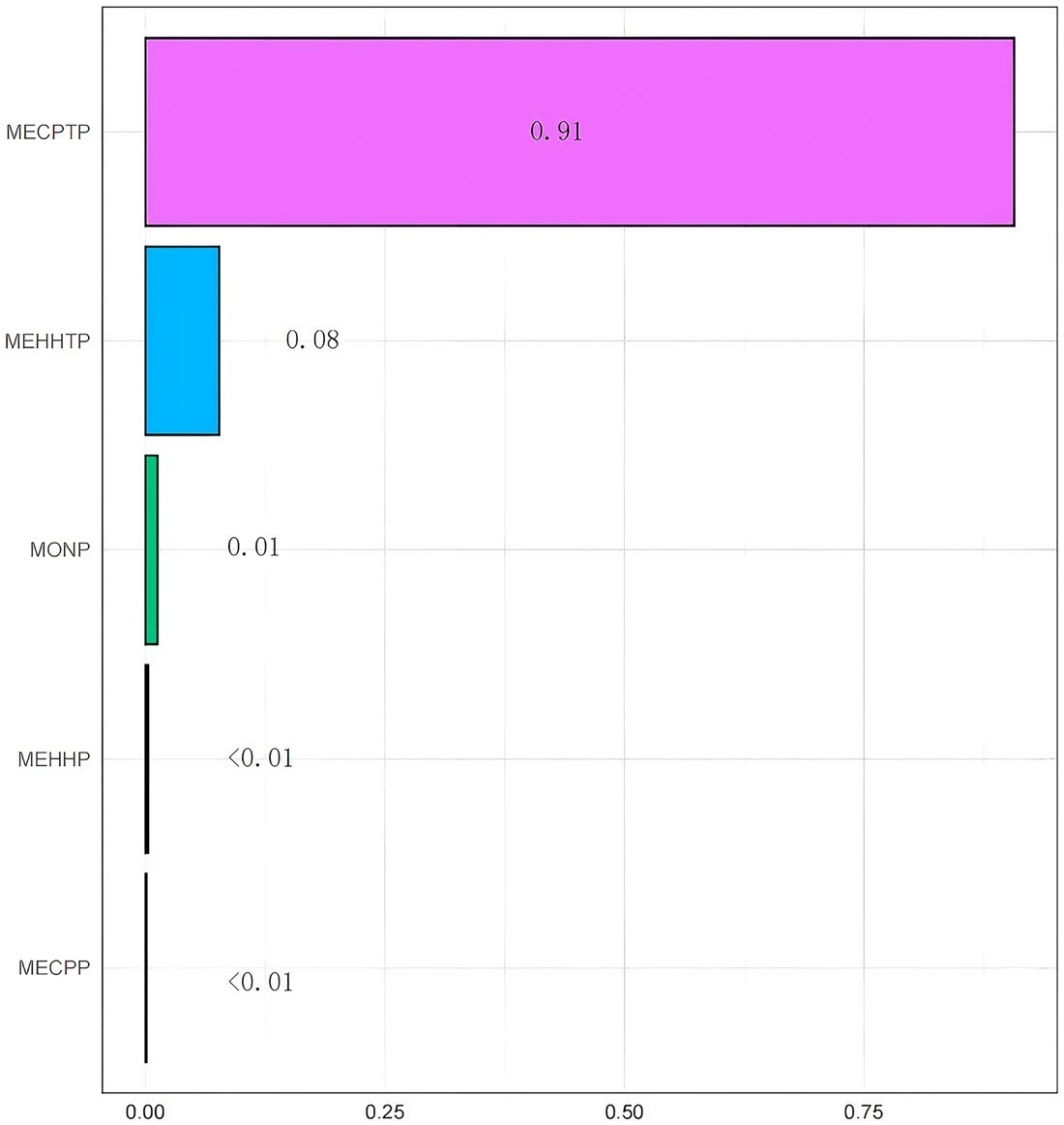
WQS regression of plasticizer exposure and eGFR. Weighted Quantile Sum (WQS) regression results showing the relative contribution of each urinary plasticizer metabolite—MEHHP and MECPP (DEHP metabolites), MEHHTP and MECPTP (DEHTP metabolites), and MONP (DiNP metabolite)—to the overall exposure index for estimated glomerular filtration rate (eGFR). Bars represent the weights assigned to each metabolite in the WQS index, reflecting their proportional influence on the combined association with eGFR. A larger weight indicates a stronger contribution to the overall mixture effect. All WQS models were constructed with 10,000 bootstrap iterations and adjusted for demographic, socioeconomic, and clinical covariates. In this analysis, MECPTP showed the predominant contribution, followed by MEHHTP, while MEHHP, MECPP, and MONP contributed minimally. Corresponding WQS weights for urinary albumin-to-creatinine ratio (UACR) are presented in Supplementary Figure S5.

The WQS regression model identified MECPTP as the predominant contributor to the overall effect on eGFR, with a weight of 0.91, indicating its dominant influence within the exposure combined. MEHHTP ranked second, with a considerably smaller weight of 0.08. MONP showed a minor contribution (weight = 0.01), while MEHHP and MECPP had negligible effects on eGFR, with weights of 0.003 and 0.001, respectively. These findings are visualized in Figure 4.

Using the Cross-Validated Ensemble of Kernels (CVEK) approach, we identified significant interactions between specific plasticizer metabolites about eGFR. MECPTP exhibited strong interactive effects with MEHHTP and MEHHP, suggesting potential synergistic or antagonistic biological interplay. No statistically significant interactions were observed among the other metabolites, indicating largely independent effects within the remaining components of the combined. The *p* values for interaction effects among metabolites are summarized in Table 3.

**Table 3.** CVEK interaction matrix between plasticizer metabolites (*p* values).

Variable	MONP	MEHHP	MECPP	MEHHTP
MEHHP	0.91			
MECPP	0.81	0.06		
MEHHTP	0.91	0.05	0.01	
MECPTP	0.50	<0.01	<0.01	0.53

Note: *p* values represent interaction significance between pairs of plasticizer metabolites based on CVEK analysis. Values < 0.05 indicate statistically significant interaction effects.

### 3.6. Sensitivity analyses

Sensitivity analyses using crude and single-pollutant models yielded results consistent with those from the fully adjusted models, reinforcing the robustness of our primary findings. Detailed estimates are provided in Table 4.

**Table 4.** Sensitivity analyses using single-pollutant and crude linear models.

Model	Variable	β	95% CI (Low)	95% CI (Up)	*p* value
Single-pollutant	MONP	−0.62	−1.86	0.61	0.32
Single-pollutant	MEHHP	−1.27	−2.67	0.14	0.08
Single-pollutant	MECPP	−1.76	−3.20	−0.31	0.02
Single-pollutant	MEHHTP	0.21	−0.69	1.11	0.65
Single-pollutant	MECPTP	0.61	−0.25	1.46	0.17
Crude model	MONP	1.44	−0.42	3.30	0.13
Crude model	MEHHP	−1.15	−3.26	0.96	0.29
Crude model	MECPP	−2.29	−4.45	−0.13	0.04
Crude model	MEHHTP	3.96	2.65	5.28	<0.01
Crude model	MECPTP	5.40	4.15	6.64	<0.01

Note: Sensitivity analysis results for associations between urinary plasticizer metabolites and eGFR using single-pollutant and crude linear regression models. CI: confidence interval.

## 3. Discussion

This study found a significant association between plasticizer metabolite levels and renal function, with evidence of a nonlinear exposure–response relationship.

Specifically, elevated MECPP and MEHHP levels (urine and blood) correlated with lower eGFR, indicating reduced kidney function. These findings are consistent with prior studies and suggest that MECPP and MEHHP may impair renal function *via* mechanisms such as oxidative stress, inflammation, and cellular injury [34]. Given their consistent links to renal outcomes, MECPP and MEHHP may be early biomarkers of environmental nephrotoxic risk.

In contrast, MEHHTP and MECPTP were positively associated with eGFR. However, elevated eGFR can indicate glomerular hyperfiltration in early nephropathy; thus, these positive associations should not be interpreted as renal protection. Rather, they may reflect compensatory filtration or hemodynamic changes that precede functional decline. Where available, albuminuria (e.g. UACR) offers complementary evidence of early injury and helps contextualize eGFR changes.

These results have important implications for evaluating plasticizer alternatives, underscoring the need to distinguish legacy compounds from newer substitutes in risk assessments. Furthermore, our study highlights complex interactions among metabolites: at higher exposures, the nephrotoxic effects of MECPP and MEHHP become more pronounced. In contrast, MEHHTP and MECPTP may have mitigating effects at certain exposure levels. Overall, these findings reinforce the link between plasticizer metabolites and renal dysfunction and offer a nuanced perspective on the varied roles of different compounds in kidney health. We used Bayesian Kernel Machine Regression (BKMR) to model high-dimensional, nonlinear exposure interactions, allowing us to capture these complex effects better. Unlike conventional linear models, BKMR can reveal synergistic or antagonistic interactions among co-occurring metabolites [32], offering a more comprehensive framework for assessing environmental health risks.

Whereas prior studies focused on individual exposures and often ignored combined effects, our analysis makes a novel contribution by systematically evaluating single-compound and joint effects. This approach broadens the current evidence base and highlights the importance of mixture–aware frameworks in future environmental epidemiology.

Plasticizers are chemical additives extensively used in consumer products, including plastics, personal care items, and medical devices. As their global use increases, plasticizers have significantly contributed to environmental pollution. Among these chemicals, DEHP has been widely used in the plastics industry for its excellent flexibility-enhancing properties. The World Health Organization classifies DEHP as an endocrine-disrupting chemical (EDC). Human exposure to DEHP can occur unintentionally *via* multiple routes, including diet, drinking water, cosmetics, and medical equipment [35]. Concerns about DEHP’s health hazards have led many countries and regions to impose regulatory restrictions on its use, accelerating the adoption of alternative plasticizers. DEHTP and DiNP have become two of the most commonly used substitutes for DEHP.DEHTP, a structural isomer of DEHP, is a versatile plasticizer used in many applications, including polymers, adhesives, sealants, children’s toys, medical devices, and food-contact materials. A U.S. Consumer Product Safety Commission survey found that DEHTP was the second most frequently detected plasticizer in 38 PVC-based children’s products [36]. Similarly, investigating of U.S. fast food items found DEHTP among the most frequently detected plasticizers in food samples [37]. DiNP, another major DEHP alternative, accounts for about 30% of the phthalate plasticizer market [38]. Market forecasts estimate that the global value of DiNP will reach USD 3.15 billion in 2022 and is projected to grow to USD 4.7 billion by 2030, with a compound annual growth rate (CAGR) of 5.3%(https://www.giiresearch.com). Given their widespread use and bioavailability, it is essential to understand the health effects of plasticizers and their metabolites—particularly their renal toxicity—to inform risk assessment and public health policy.

Plasticizers’ nephrotoxic potential is closely tied to their metabolic behavior in the body. These chemicals can enter the body *via* ingestion, inhalation, or dermal absorption. Chronic exposure can lead to bioaccumulation and disrupt multiple organ systems, especially the kidneys. Because the kidneys filter blood and reabsorb many substances, they are particularly vulnerable to xenobiotics, including plasticizer metabolites. These metabolites can bind to intracellular receptors, leading to tubular dysfunction, increased oxidative stress, and interstitial fibrosis in renal tissue [39]. Exposure to plasticizer metabolites has been linked to various adverse outcomes, including endocrine disruption, immune dysregulation, and metabolic disorders [40].

DEHP undergoes hydrolysis in renal tissue [41], causing structural changes in glomeruli and tubules and elevating biomarkers such as blood urea nitrogen (BUN) and serum creatinine [42–44]. In mice, DEHP exposure promotes renal inflammation, fibrosis, and damage to mitochondria and the endoplasmic reticulum [45,46]. These effects may be mediated by oxidative stress and activation of the p38 MAPK/NF-κB pathway, thereby exacerbating diabetic nephropathy [39]. Animal studies have further confirmed DEHP’s nephrotoxic effects. Prenatal DEHP exposure impairs placental development in mice, leading to lower birth weight, premature delivery, and higher fetal mortality [47]. Similarly, high-dose dermal exposure to DiNP induces oxidative renal damage in mice, evidenced by elevated ROS, malondialdehyde (MDA), and DNA–protein crosslinks (DPC) in the kidneys—suggesting a nephrotoxic risk [48].

Although DEHP and its metabolites are well studied, evidence regarding the nephrotoxicity of its replacement, DEHTP, is limited. Despite DEHTP’s wide use as a next-generation plasticizer, its toxicological profile remains poorly characterized. No epidemiological data link DEHTP metabolites to renal function markers such as eGFR. Clinical and population-level assessments of DEHTP’s renal risks are sparse, and mechanistic insights are lacking. Therefore, future studies should systematically evaluate DEHTP’s nephrotoxic potential under environmentally relevant exposure levels to fill current knowledge gaps.

These findings underscore the multiple mechanisms plasticizers may impair renal function, including oxidative stress, inflammation,autophagic dysregulation, and ionic imbalance. Continued research is essential to clarify chronic plasticiser exposure’s long-term health risks and inform evidence-based regulations and public health interventions.

Beyond the kidneys, plasticizers can adversely affect multiple other organ systems. Recent evidence links internal DEHTP exposure to an increased risk of non-alcoholic fatty liver disease (NAFLD) [49]. Animal studies show that DEHTP can disrupt thyroid and sex hormone signaling at various developmental stages [50].

Phthalate exposure is well documented to have anti-androgenic effects on the male reproductive system. These effects likely occur by disrupting testosterone synthesis and hormonal homeostasis, ultimately impairing male reproductive health. High phthalate exposure has been linked to reduced semen quality, lower sperm count and motility, and decreased testosterone levels—factors that may increase infertility risk [51].

The nervous system is another vulnerable target of plasticizer toxicity. Prenatal exposure to some plasticizers has been linked to impaired fetal brain development, resulting in long-term neurobehavioral consequences like reduced cognitive function, attention deficits, and behavioral abnormalities in children [52]. Overall, these findings suggest that plasticizer exposure poses a multisystem health threat, affecting the kidneys, liver, endocrine and reproductive systems, and neurodevelopment. Future research should elucidate the underlying toxicological mechanisms and assess the long-term health consequences of chronic plasticizer exposure. Such evidence is needed to inform more decisive regulatory actions and reduce the population-level burden of plasticizer-related disease. Mitigating plasticizer exposure requires both stringent regulation of environmental sources and greater public awareness of the associated health risks. Policymakers should prioritize enforcing plasticizer restrictions, especially in high-exposure consumer products (e.g. food packaging, children’s toys, personal care items). Responding to evidence of harm, many U.S. states and European bodies have enacted laws limiting specific phthalates (e.g. DNOP, DIDP, DINP, BBP, DBP, DEHP), with focusing reducing exposure in children’s products [53].

Although emerging alternatives like MEHHTP and MECPTP may have lower acute toxicity, our findings underscore the urgent need for comprehensive long-term safety evaluations. Without rigorous toxicological assessment, the widespread adoption of substitutes could lead to unforeseen health consequences. To ensure progress in chemical safety, precautionary regulations must be guided by robust evidence and continually updated to reflect evolving environmental exposures.

This study has several limitations to consider. First, the cross-sectional design precludes establishing causality between plasticizer metabolite exposure and renal function. Additionally, unmeasured confounders (e.g. diet, medication use, and other lifestyle factors) may have biased the observed associations. Second, although we used high-quality, nationally representative NHANES 2015–2018 data, the findings may not generalize to other populations. Future studies should use more recent and geographically diverse datasets to capture current exposure profiles and global variability.

Finally, assessing plasticizer exposure *via* urinary metabolites is prone to measurement error and reflects only short-term exposure due to the compounds’ rapid metabolism and excretion. More precise, integrated exposure assessment methods (e.g. longitudinal biomonitoring, repeated sampling, and combining environmental and dietary data) are needed to capture long-term exposure patterns and strengthen causal inference.

In summary, our findings link urinary plasticizer metabolites to reduced renal function, suggesting that chronic exposure to these chemicals may elevate the risk of chronic kidney disease and other renal disorders. Although our findings provide valuable epidemiological evidence, the cross-sectional design limits causal inference. Longitudinal studies are needed to clarify the long-term impacts of plasticizer metabolite exposure on kidney function.

Mechanistically, plasticizers may cause nephrotoxic effects by binding to intracellular receptors and triggering oxidative stress, inflammation, and renal fibrosis—insights point to potential pathways for further research. Moreover, as alternatives like DEHTP see wider use, future studies should evaluate their long-term safety and examine interactions with other environmental toxicants to ensure these substitutes do not introduce new health risks.

## 5. Conclusion

Collectively, our findings offer critical evidence linking plasticizer exposure to renal impairment. Continued investigation into these exposures’ biological mechanisms and broader health consequences will be essential for informing science-based public health policies and chemical regulation frameworks.

## Supplementary Material

Supplemental Material

Figure S2.jpg

Supplementary Figure 1.pdf

Figure S3.jpg

Figure S4.jpg

Figure S5.jpg

## Data Availability

The datasets generated and analyzed during the current study are publicly available from the NHANES database (https://www.cdc.gov/nchs/nhanes/). Additional data used in this study are available from the corresponding author on reasonable request.

## Abbreviations:

PAEs: Phthalates
DEHP: Di(2-ethylhexyl) phthalate
DEHTP: Di(2-ethylhexyl) terephthalate
DiNP: Diisononyl phthalate
MEHHP: Mono(2-ethyl-5-hydroxyhexyl) phthalate
MECPP: Mono(2-ethyl-5-carboxypentyl) phthalate
MEHHTP: Mono(2-ethyl-5-hydroxyhexyl) terephthalate
MECPTP: Mono(2-ethyl-5-carboxypentyl) terephthalate
MONP: Monoisononyl phthalate
CKD: Chronic kidney disease
eGFR: Estimated glomerular filtration rate
NHANES: National Health and Nutrition Examination Survey
BKMR: Bayesian Kernel Machine Regression
WQS: Weighted quantile sum regression
MDRD: Modification of diet in renal disease
CKD-EPI: Chronic Kidney Disease Epidemiology Collaboration
HPLC-ESI-MS/MS: High-performance liquid chromatography–electrospray ionization–tandem mass spectrometry
PIR: Poverty income ratio
BMI: Body mass index
LOD: Limit of detection
SD: Standard deviation
PIP: Posterior inclusion probability
CVEK: Cross-validated ensemble of kernels
MCMC: Markov Chain Monte Carlo
HbA1c: Hemoglobin A1c
BUN: Blood urea nitrogen
ROS: Reactive oxygen species
NF-κB: Nuclear factor kappa-light-chain-enhancer of activated B cells
AMPK: AMP-Activated protein kinase
ULK1: Unc-51 Like Autophagy activating kinase 1
AQP: Aquaporin
ATPase: Adenosine triphosphatase
