# Supplementary material for: Urinary and circulatory levels of plasticizer metabolites and their associations with renal function: a cross-sectional analysis of the NHANES cohort
Source: Ann Med. 2025 Oct 5;57(1):2559125. doi: 10.1080/07853890.2025.2559125 (PMC12502113; doi:10.1080/07853890.2025.2559125)
Supplement: Supplemental Material [file IANN_A_2559125_SM5474.docx]

**Supplementary Figure Caption**

**Figure S2**. Spearman correlation matrix among urinary plasticizer metabolites, eGFR, and UACR Spearman correlation coefficients are presented to illustrate the pairwise relationships among five urinary plasticizer metabolites—MEHHP and MECPP (DEHP metabolites), MEHHTP and MECPTP (DEHTP metabolites), and MONP (DiNP metabolite)—and two renal outcomes: estimated glomerular filtration rate (eGFR) and urinary albumin-to-creatinine ratio (UACR). The strength and direction of the associations are indicated by the color scale and the magnitude of the correlation coefficient. Statistical significance is denoted by P values, with ***P < 0.001, **P < 0.01, and *P < 0.05.

**Figure S3.** Restricted cubic spline (RCS) curves for associations of urinary plasticizer metabolites with UACR Restricted cubic spline models depicting potential nonlinear exposure –response relationships between each metabolite and UACR. The solid red line represents the estimated association, while the shaded gray area indicates the 95% confidence interval (CI). The P value for non-linearity is displayed in each panel. Models were adjusted for the same covariates as the main analyses, and metabolite concentrations were log ₁ ₀ -transformed prior to modeling. Results complement Figure 2, which presents analogous curves for eGFR.

**Figure S4**. BKMR analysis for UACR: single- and joint-exposure effects of urinary plasticizer metabolites Bayesian Kernel Machine Regression (BKMR) results showing the associations of five urinary metabolites with UACR. (A) Univariate exposure–response functions, obtained with the other metabolites fixed at their medians, display posterior mean estimates (solid line) with 95% credible intervals (shaded bands). (B) Bivariate exposure–response functions, with one metabolite varied while fixing another at the 25th, 50th, and 75th percentiles, illustrate pairwise relationships; the remaining metabolites were held constant at medians. (C) Overall mixture effect, estimated as the predicted change in UACR when simultaneously shifting all metabolites across exposure percentiles relative to the median mixture. (D) Single-exposure effects under different mixture backgrounds, with the other four metabolites fixed at the 25th, 50th, or 75th percentile. All models were adjusted for relevant covariates and run with 10,000 MCMC iterations. Posterior Inclusion Probabilities (PIPs) for UACR are reported in the main text.

**Figure S5.** Weighted Quantile Sum (WQS) regression weights for the association between plasticizer mixture and UACR Weighted Quantile Sum (WQS) regression results quantifying the relative contributions of five urinary metabolites to the overall exposure index for UACR. Bars represent the weights assigned to each metabolite, with higher values reflecting stronger contributions to the mixture–UACR association. WQS indices were constructed using 10,000 bootstrap iterations, with covariate adjustment consistent with the main models. In this analysis, MECPTP and MEHHTP contributed the most to the overall index, whereas MECPP, MEHHP, and MONP exhibited minimal contributions. For comparison, WQS regression weights for eGFR are presented in Figure 4.
