# Supplementary figures and images for: Urinary and circulatory levels of plasticizer metabolites and their associations with renal function: a cross-sectional analysis of the NHANES cohort
Source: Ann Med. 2025 Oct 5;57(1):2559125. doi: 10.1080/07853890.2025.2559125 (PMC12502113; doi:10.1080/07853890.2025.2559125)

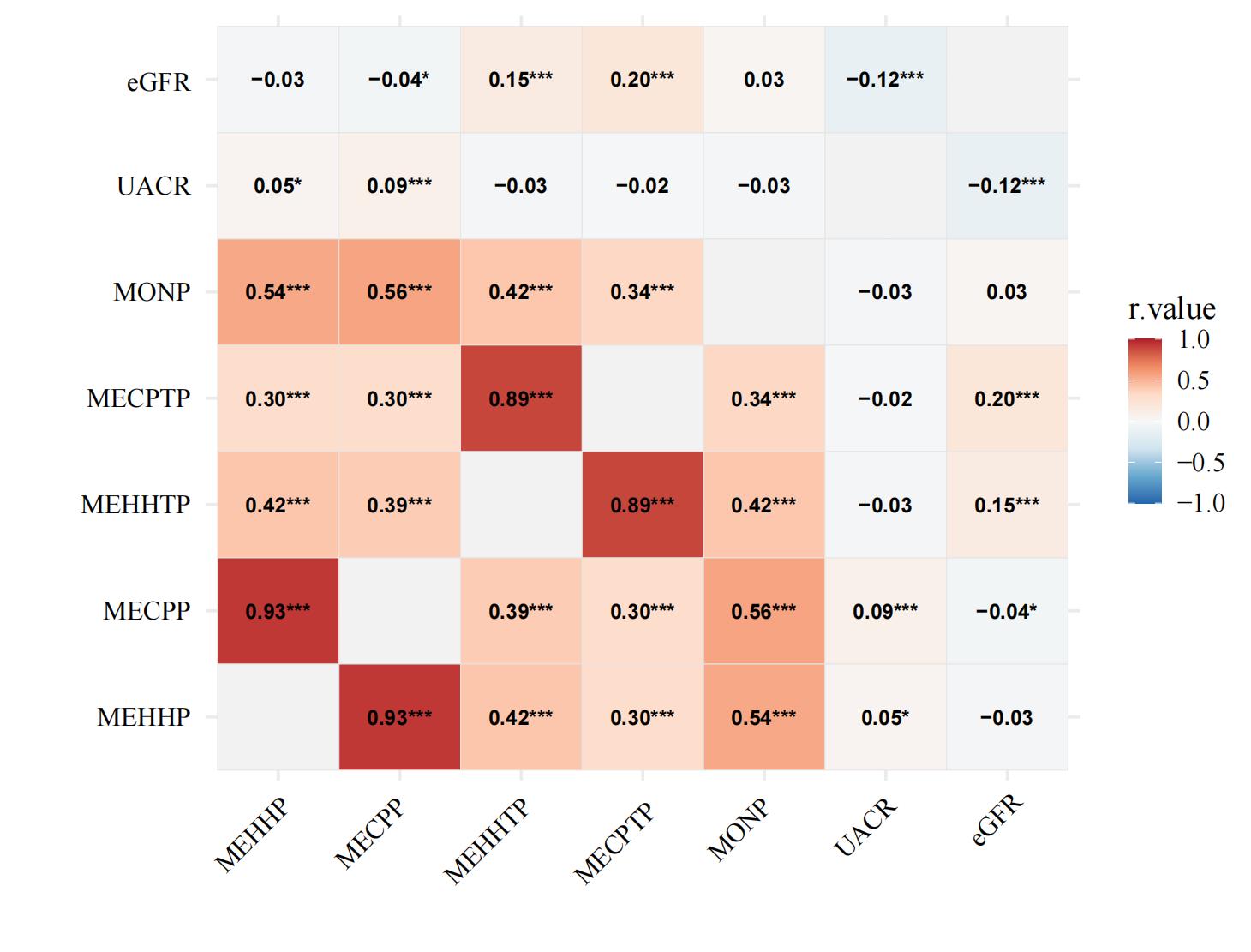

Supplement: Figure S2.jpg [file IANN_A_2559125_SM0680.jpg]

## Supplementary Figure 1

Flowchart of participant selection from NHANES 2015–2018.

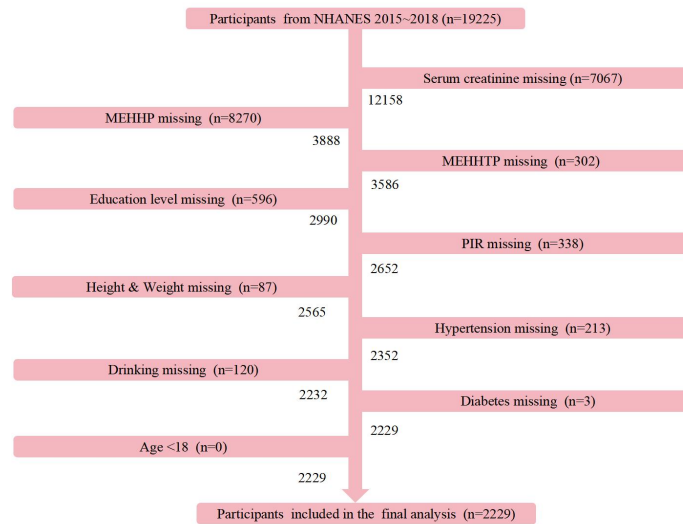

Supplement: Supplementary Figure 1.pdf [file IANN_A_2559125_SM0679.pdf]

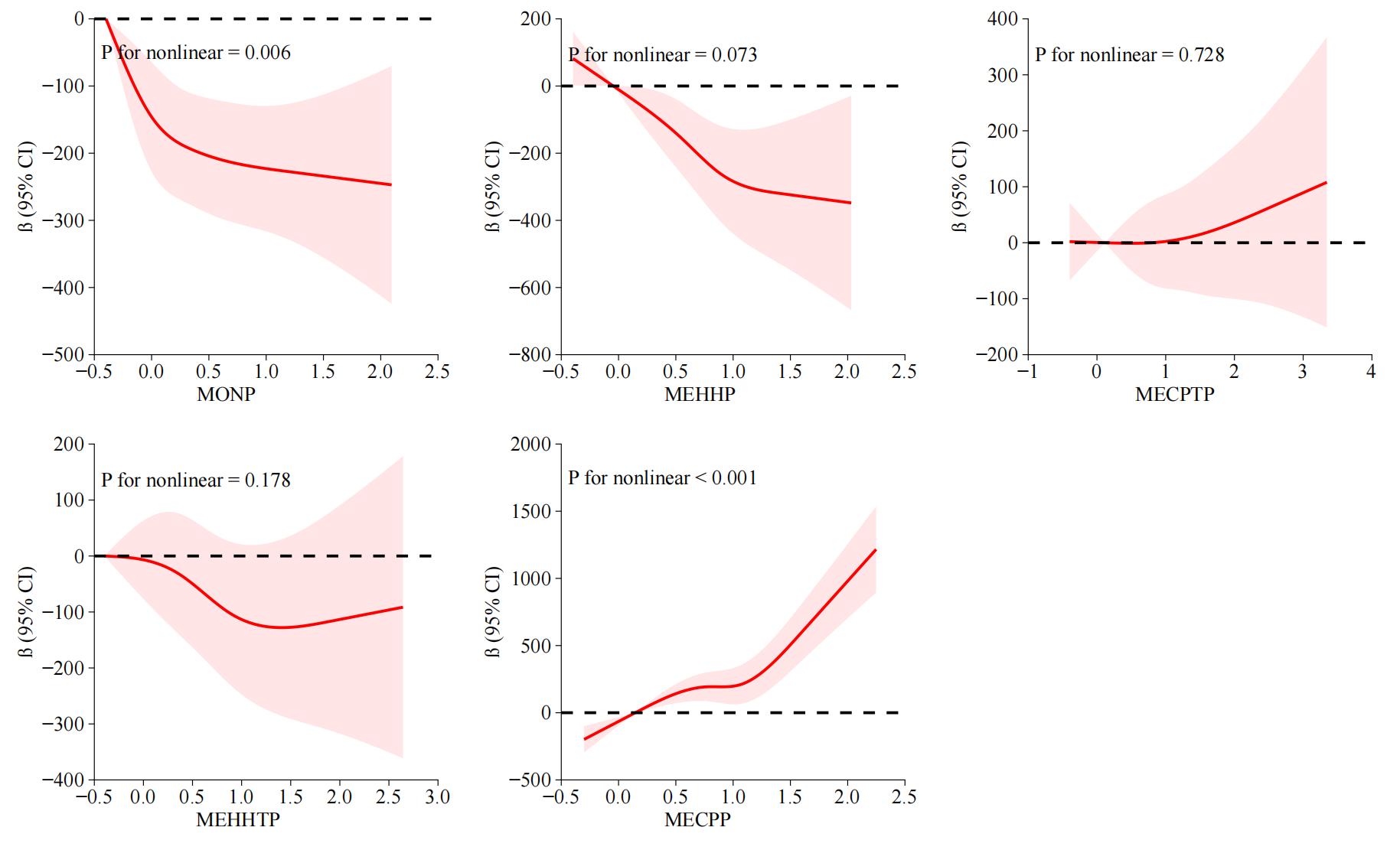

Supplement: Figure S3.jpg [file IANN_A_2559125_SM0678.jpg]

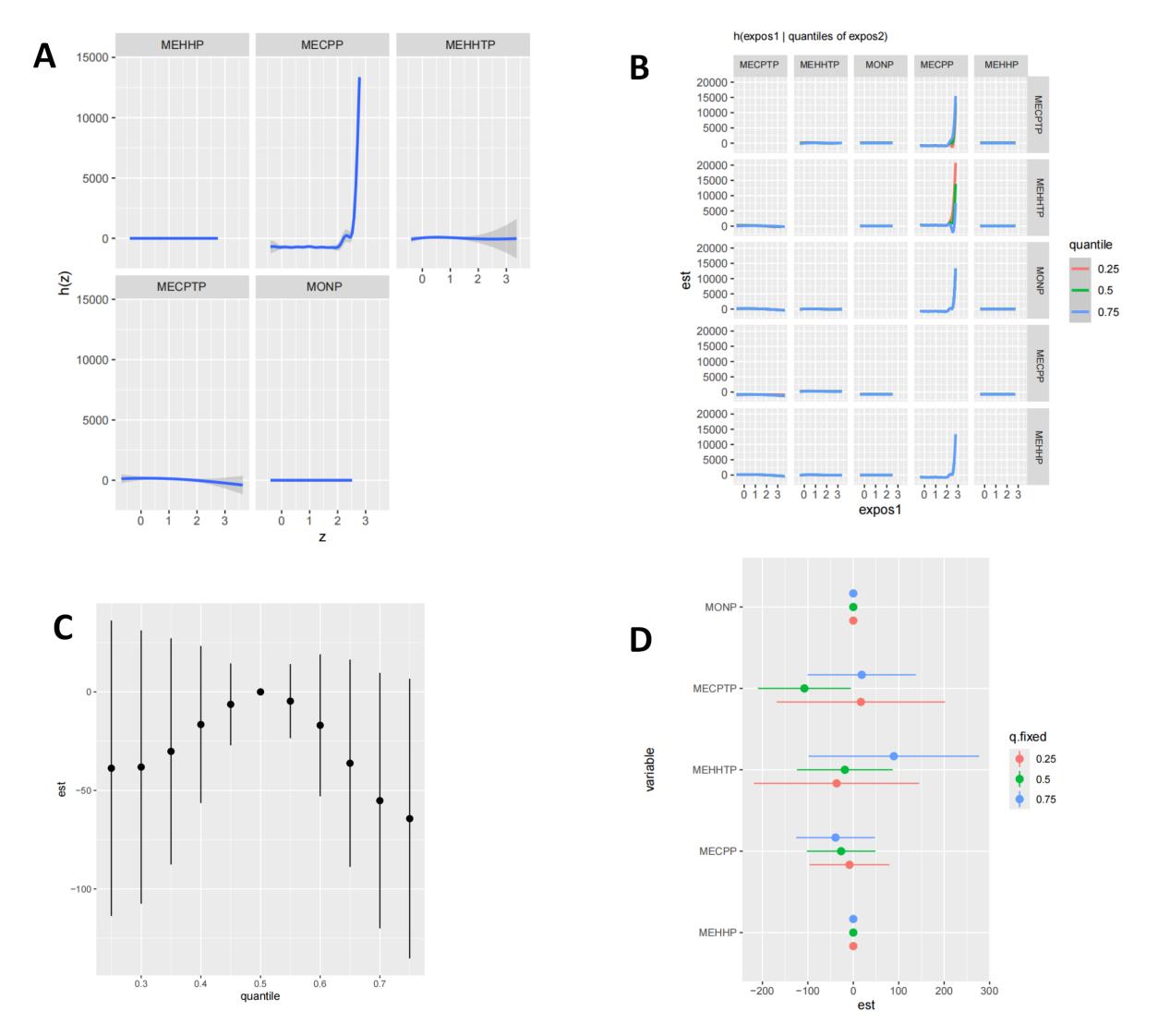

Supplement: Figure S4.jpg [file IANN_A_2559125_SM0677.jpg]

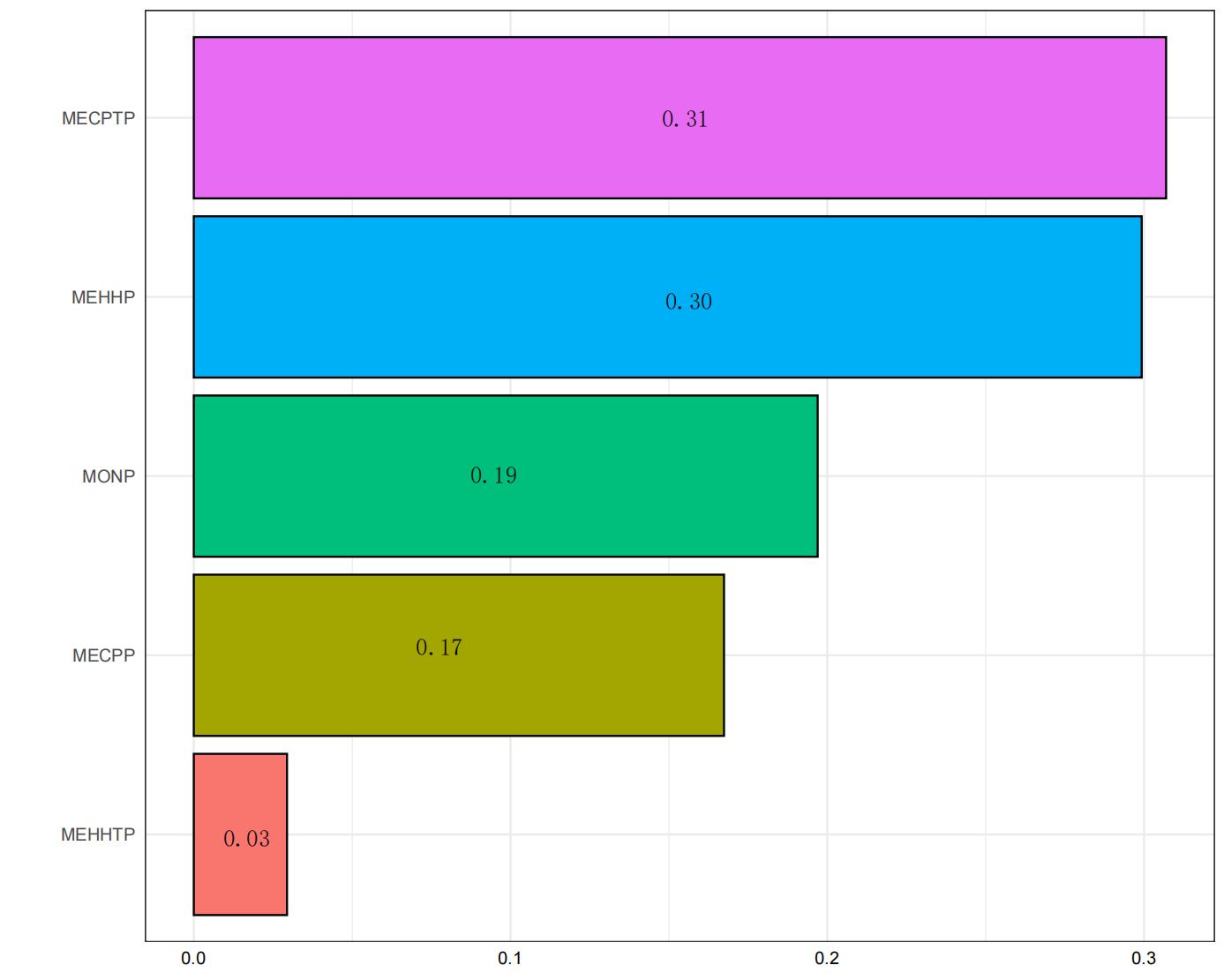

Supplement: Figure S5.jpg [file IANN_A_2559125_SM0676.jpg]
